# Skin Photoprotection and Anti-Aging Benefits of a Combination of Rosemary and Grapefruit Extracts: Evidence from In Vitro Models and Human Study

**DOI:** 10.3390/ijms26094001

**Published:** 2025-04-23

**Authors:** Pau Navarro, Julián Castillo, Jonathan Jones, Adrián García, Nuria Caturla

**Affiliations:** 1 Research and Development Department, Monteloeder SL, Miguel Servet 16, 03203 Elche, Spain; paunavarro@monteloeder.com (P.N.); jonathanjones@monteloeder.com (J.J.); adriangarcia@monteloeder.com (A.G.); 2 Food Technology & Nutritional Department, Universidad Católica San Antonio de Murcia (UCAM), Campus de los Jerónimos, Carretera Guadalupe, 30107 Murcia, Spain; jcsanchez@ucam.edu

**Keywords:** *Rosmarinus officinalis*, *Citrus paradisi*, photoaging, photoprotection, erythema, dietary supplement, collagen, elastin, metalloproteinases and clinical study

## Abstract

Skin exposure to ultraviolet radiation (UVR) causes oxidative stress, inflammation, and collagen degradation and can trigger erythema. While topical formulas protect the skin from UV damage, there is growing evidence that certain botanical ingredients taken orally may have an added benefit. This study evaluated the photoprotective, anti-photoaging, and anti-erythema efficacy of a combination of rosemary and grapefruit extract (Nutroxsun^®^). Radical oxygen species (ROS) generation and interleukin production were determined in UV-irradiated keratinocytes (HaCaT). Also, collagen and elastin secretion and metalloproteinase (MMP-1 and MMP-3) content were assessed in UV-irradiated fibroblasts (NHDFs). Furthermore, a placebo-controlled, randomized, crossover study was conducted in 20 subjects (phototypes I to III) receiving two doses, 100 and 200 mg, of the ingredient. Skin redness (a* value, CIELab) after exposure to one minimal erythemal dose of UVR was assessed. As a result, the botanical blend significantly attenuated the UVR-induced reductions of procollagen I and elastin and lowered MMP-1 and MMP-3 protein secretion. Also, a reduction in ROS and proinflammatory interleukins (IL-1, IL-8, and IL-6) was observed. Finally, the botanical blend, at both doses, significantly reduced UV-induced erythema reaction from the first day of intake and accelerated recovery. These findings reinforce the potential of this ingredient as an effective dietary solution to protect the skin against UV-induced damage.

## 1. Introduction

The skin is continually exposed to various environmental stressors, with ultraviolet radiation (UVR) being one of the most significant contributors to both acute and chronic skin damage. While moderate sun exposure is essential for vitamin D synthesis, immune modulation, nitric oxide release, and excessive UV exposure significantly affect skin health, leading to acute reactions such as sunburn and longer-term consequences like photoaging and even skin cancer [1,2]. UVR, particularly UVB (290–320 nm) and UVA (320–400 nm), penetrates the skin and induces the production of reactive oxygen species (ROS), causing oxidative stress, inflammation, and the degradation of key structural proteins [3]. The short-term effects of sun exposure include sunburn, which is primarily characterized by erythema (redness) and is mediated by the release of neuropeptides, histamine, prostaglandins, serotonin, and ROS [4,5], as well as the upregulation of various pro-inflammatory cytokines such as tumor necrosis factor-alpha (TNF-α), IL-1, IL-6, and IL-8 [6,7]. Additionally, lipid peroxidation generates reactive compounds like malondialdehyde, which further contribute to cellular damage and may induce DNA alterations, increasing the risk of skin cancers [8]. Prolonged or repetitive exposure to UV radiation can lead to immune suppression and increase the risk of developing cancer through the production and secretion of different cytokines [9,10]. Also, chronic UV exposure is closely linked to photoaging, a process that accelerates the development of wrinkles, loss of skin elasticity, and hyperpigmentation [3]. Numerous mechanisms are involved in UV-induced photodamage, with ROS playing a central role [11]. ROS not only induce DNA damage but also influence cell signaling pathways, leading to an imbalance in the skin’s antioxidant defenses, which accelerates photodamage [3,12]. Specifically, ROS activate transcription factors, such as AP-1 and nuclear factor-kappa B (NF-κB), which increase the expression of matrix metalloproteinases (MMPs). These enzymes degrade key extracellular matrix (ECM) components such as collagen and elastin, compromising skin integrity [11,12]. In addition, ROS upregulate the expression of inflammatory mediators, such as IL-1, IL-6, IL-8, and TNF-α, while downregulating the cytokine transforming growth factor beta (TGF-β) and Smad signaling pathways [13], thereby impairing the skin matrix [12]. Moreover, ROS contribute directly or indirectly to UV-induced mitochondrial apoptosis, amplifying cellular damage [14].

One of the main mechanisms underlying dermal atrophy is the progressive decline in ECM components, including collagens, elastin, glycosaminoglycans, and hyaluronic acid. Collagen fibers are a major component of the ECM, accounting for 75% of the dry weight of skin and providing tensile strength and elasticity [12]. Together with elastin, collagen plays a major role in the skin’s appearance. Most components of the skin, including collagen, undergo continuous turnover [12]. New collagens are continually produced and recycled throughout life. At a younger age, the synthesis of collagen predominates, whereas after about the age of 40, the degradation of collagen accelerates due to the increased activity of MMP enzymes [12,15]. MMPs are a group of zinc-containing endopeptidases that participate in various physiological functions in the human body, including tissue remodeling and degradation of proteins in the extracellular matrix.

MMPs exhibit substrate specificity [16], and those targeting key dermal components like collagen I and III, elastin, and minor collagens (V, VI, XII) are particularly important for skin structure and regeneration [17,18]. While at least 23 different subtypes of MMP enzymes have been identified in humans, only a few of them have a significant impact on the skin matrix and are known to be associated with skin aging: MMP-1, 2, 3, 8, 9, 12, and 13 [16,18,19]. Studies have revealed that UVR particularly elevates at least three different MMPs in human skin in vivo, i.e., interstitial collagenase (MMP-1), stromelysin-1 (MMP-3), and gelatinase (MMP-9) [20].

Dietary approaches to photoprotection have recently gained attention, with a particular focus on plant polyphenols due to their ability to mitigate UVR-induced skin damage. Indeed, several vitamins, minerals, carotenoids, and polyphenols from botanical sources have been proven effective to help prevent sunburn and skin damage, and contribute to life-long protection against the damaging effects of UVR [21,22,23,24]. Previous research has demonstrated that rosemary and grapefruit extracts contain phenolic compounds that work synergistically to scavenge free radicals generated by solar radiation [25]. Building on these findings, Nutroxsun^®^ was developed as a food supplement ingredient combining rosemary (*Rosmarinus officinalis*) and grapefruit (*Citrus paradisi*) extracts [25,26], with the aim of enhancing the skin’s natural defense mechanisms against UV-induced oxidative stress. In a double-blind, randomized, parallel-group study involving 90 participants over two months, the tested ingredient was shown to significantly increase the minimum erythema dose (MED), reduce skin lipid peroxidation caused by UVB and UVA exposure, and improve skin wrinkles and elasticity [26]. In the present study, we aimed to further investigate the photoprotective and anti-photoaging efficacy of Nutroxsun^®^ through both in vitro and in vivo models. Specifically, we evaluated the ability of the blend in vitro to reduce ROS and proinflammatory cytokine production and preserve the skin matrix proteins in response to UVR. Additionally, a placebo-controlled clinical trial was conducted to assess the efficacy of the supplement in reducing UV-induced erythema and accelerating the skin’s recovery from sunburn. This integrative approach aims to validate Nutroxsun^®^ as a dietary strategy for continuous photoprotection, offering both short- and long-term skin health benefits.

## 2. Results

### 2.1. MMP Inhibition Study

The ability of Nutroxsun^®^ to inhibit different metalloproteinases was studied enzymatically in vitro. The metalloproteinases studied and their substrates are listed in Table 1.

**Figure 1:**
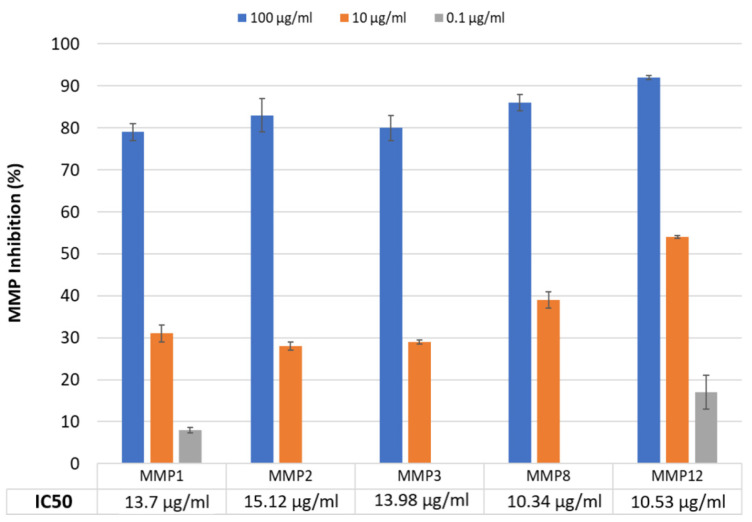
Inhibition profile of Nutroxsun^®^ on matrix metalloproteinases (MMPs). The inhibitory effect was measured using a fluorometric assay, and the results are expressed as the percentage inhibition of enzymatic activity. Each bar represents the mean ± SD of two experiments. IC50 values, obtained with the ATT Bioquest online tool, are indicated for each MMP.

The results for the MMP inhibition profile are presented in Figure 1. Nutroxsun^®^ inhibited all the tested MMPs in a dose-dependent manner. The half maximal inhibitory concentration (IC50) values for all MMPs were below 20 µg/mL, with the most pronounced inhibitory effect observed in MMP-12, which had an IC50 of 10.53 µg/mL. No significant differences were observed among the different MMPs (*p* > 0.05).

### 2.2. Regulation of MMP-1 and MMP-3 Secretion by Nutroxsun^®^ in UV-Irradiated and Non-Irradiated Human Dermal Fibroblasts

MMP-1 and MMP-3 are known to degrade collagen and elastin fibers. MMP-1, an interstitial collagenase, can degrade native fibrillar collagen types I, II, III, IX, and XI. MMP-3, or stromelysin-1, has a broad substrate specificity that includes casein, proteoglycans, fibronectin, elastin, and laminin, as well as collagen types III, IV, V, IX, and X [17,27,28]. Also, MMP-3 may activate other MMPs necessary for tissue remodeling, including MMP-1, -7, and -9 [29]. Cooperative actions of MMP-1 and MMP-3 further augment the final proteolytic action [30].

**Figure 2:**
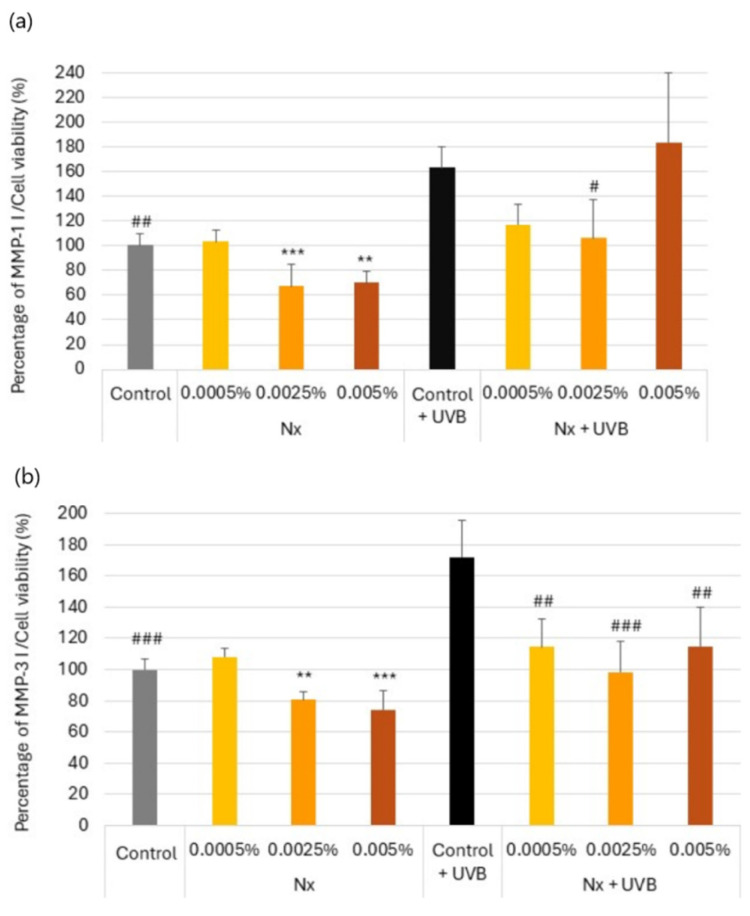
Bar graph representing MMP-1 (**a**) and MMP-3 (**b**) content in NHDF culture supernatants, normalized to cell viability in the presence of Nutroxsun^®^ (Nx) and upon exposure to UVR, as indicated in Section 4. The graphs are plotted as the percentage of change in MMP content compared to the non-irradiated untreated control at 100%. Data are expressed as mean ± SD. ** (*p* < 0.01) and *** (*p* < 0.001) indicate statistically significant differences compared to the non-irradiated untreated control (gray bars) and # (*p* < 0.05), ## (*p* < 0.01) and ### (*p* < 0.001) indicate statistically significant differences compared to the UVR-untreated controls (black bars).

ELISA was conducted to determine the inhibitory effects of Nutroxsun^®^ on MMP-1 and MMP-3 secretion in UV-irradiated and non-UV-irradiated NHDFs. As shown in Figure 2, Nutroxsun^®^ inhibited both MMP-1 and MMP-3 secretion.

In the absence of UVR, the normalized secreted levels of MMP-1 showed a statistically significant dose-dependent decrease with Nutroxsun^®^, reducing by 32.5% and 29.8% at concentrations of 0.025% and 0.005%, respectively (Figure 2a). Consistently, MMP-3 reproduced the same pattern, with a statistically significant decrease in MMP-3 levels by 19.2% and 25.7% at 0.025% and 0.005%, respectively (Figure 2b).

Upon exposure to UVB, data showed that MMP-1 and MMP-3 were upregulated by 72.2% and 63.4%, respectively, compared to non-irradiated controls. Regarding MMP-1, Nutroxsun^®^ at 0.0025% significantly counteracted the UVB-induced upregulation of MMP-1 by 57.2% versus the untreated Control + UVB (Figure 2a). MMP-3 behaved quite similarly, with a statistically significant inhibition driven by Nutroxsun^®^ at the three dosages tested (Figure 2b).

### 2.3. Effect of Nutroxsun^®^ on the Secretion of Elastin and Procollagen Proteins in UVR-Exposed Human Dermal Fibroblasts

Photoaging is caused by repeated exposure to UVR as a consequence of the reduction in collagen and elastin production [12,31,32]. Type I collagen is the most abundant protein in the ECM, representing 80–90% of skin collagen. Fibroblasts secrete procollagen fiber into the ECM, where they form larger collagen bundles. Collagen provides the support matrix underpinning healthy skin and is a key determinant for the preservation of skin firmness and elasticity [15,33]. Fibroblasts also produce elastin, which gives the skin flexibility to stretch by facilitating long-range deformability, followed by a recoil to allow tissues to return to their original conformation. This is a critical functionality for maintaining skin elasticity and resilience [31,32].

**Figure 3:**
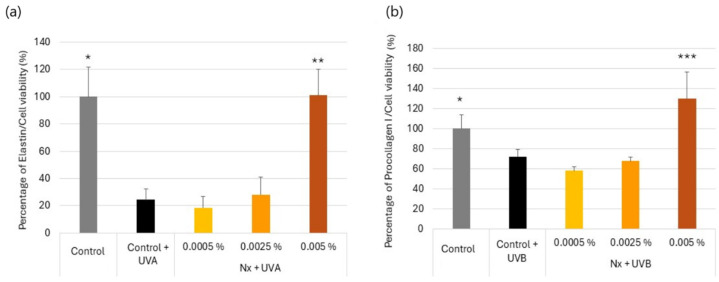
Bar graph representing elastin (**a**) and pro-collagen I (**b**) content in NHDF culture supernatants, normalized to cell viability in the presence of Nutroxsun^®^ (Nx) and upon exposure to UVR, as indicated in Section 4. The graphs are plotted as the percentage of change in protein content compared to the non-irradiated, untreated control at 100%. Data are expressed as mean ± SD. * (*p* < 0.05), ** (*p* < 0.01), and *** (*p* < 0.001) indicate statistically significant differences compared to UVR-untreated controls (black bars).

Thus, we investigated the potential effect of Nutroxsun^®^ in procollagen and elastin production when exposed to UV light (Figure 3). UV exposure significantly reduced pro-collagen I levels by 26.0% compared to the non-irradiated control (Figure 3b), whereas elastin significantly reduced by 75.5% upon exposure to UVA radiation compared to the non-irradiated control (Figure 3a). On the other hand, the presence of Nutroxsun^®^ at the higher concentrations significantly increased the presence of both elastin and procollagen compared to UV-irradiated untreated control cells.

### 2.4. Impact of Nutroxsun^®^ on ROS Generation in UVB-Irradiated Keratinocytes

**Figure 4:**
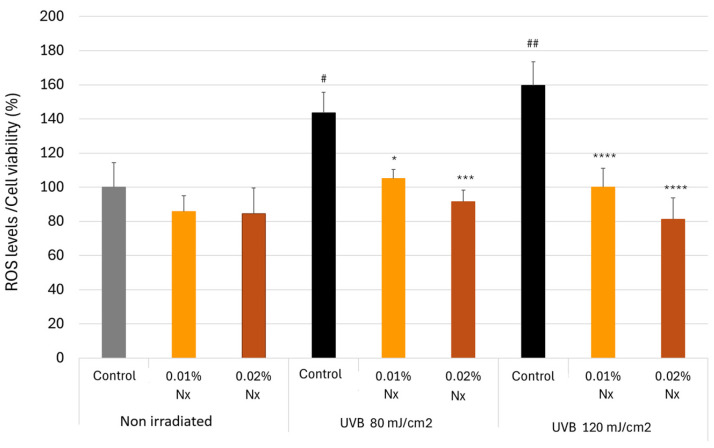
Intracellular ROS generation in HaCaT cells in the presence of Nutroxsun^®^ (Nx) and normalized to cell viability. Keratinocytes were treated with Nx (0.01% or 0.02%) and exposed to UVB radiation (80 m or 120 mJ/cm^2^). The graph is plotted as the percentage of change in ROS production compared to the non-irradiated untreated control at 100% (gray bar). Data are expressed as mean ± SD. * (*p* < 0.05), *** (*p* < 0.001), and **** (*p* < 0.0001) indicate significant differences compared to their respective untreated control cells. # (*p* < 0.05) and ## (*p* < 0.001) indicate statistically significant differences compared to the untreated, non-irradiated control (gray bar).

UVB radiation has been reported to induce the first stage of intracellular ROS, such as superoxide radical anions (O2•), H_2_O_2_, and hydroxyl radicals (•OH), either directly or through enzymatic activation [34,35]. To evaluate whether the photoprotective effect of Nutroxsun^®^ was due to its free radical scavenging capacity, we assessed its efficacy in reducing intracellular ROS generation induced by UVB radiation. Cells were exposed to 80 mJ/cm^2^ or 120 mJ/cm^2^ UVB in the presence of 0.01% and 0.02% Nutroxsun^®^. Our results, using the fluorescent probe H_2_DCFDA, which is sensitive to the aforementioned ROS and most likely to •OH [36], indicated that Nutroxsun^®^ decreased the intracellular UVB-induced ROS levels in a dose-dependent manner (Figure 4).

As shown, after irradiation with UVB, ROS levels increased significantly by 43.6% (*p* < 0.0001) and 59.5% (*p* < 0.0001) in cells irradiated with 80 or 120 mJ/cm^2^ doses, respectively, compared to non-irradiated untreated controls. In contrast, when cells were incubated with Nutroxsun^®^ during UVB exposure, the increase in ROS production significantly reduced at both concentrations, independently of the UVB intensity. When the cells were exposed to 120 mJ/cm^2^ UVB radiation and cultured with Nutroxsun^®^ 0.02%, ROS production was completely inhibited (Figure 4).

### 2.5. Impact of Nutroxsun^®^ on Interleukin Generation in UVB-Irradiated Keratinocytes

**Figure 5:**
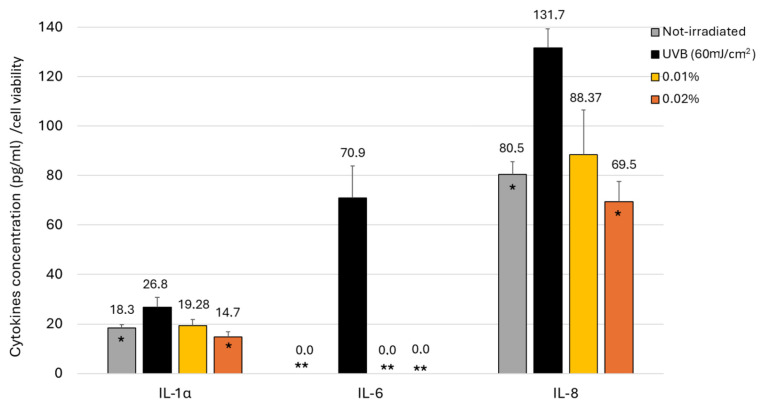
Bar graphs represent the concentration, in pg/mL, of IL-1α, IL-6 and IL-8 in the cultured media and normalized to cell viability in HaCaT treated with 0.01% and 0.02% of Nutroxsun^®^ (Nx) and exposed to UVB radiation (60 mJ/cm^2^). The gray bars represent the non-irradiated untreated cells (control). Data are expressed as mean ± SD. * (*p* < 0.05) and ** (*p* < 0.01) indicate significant differences compared to UVB-untreated controls (black bars).

Keratinocytes are the main producers of cytokines in the epidermis, with interleukins being the most abundant pro-inflammatory compounds produced when exposed to UVR (IL-1 α, IL-3, IL-6, IL-8, and IL-33) [37,38]. Subsequently, the concentrations of cytokines IL-1α, IL-6, and IL-8 were determined in UVB-irradiated keratinocytes exposed to the botanical blend (Figure 5). Specifically, cytokines IL-1α, IL-8, and IL-6 were detected in the supernatants of cultured keratinocytes 24 h after UVB exposure (60 mJ/cm^2^). Significant differences were obtained between the non-irradiated and irradiated controls (Figure 5). A significant reduction (*p* < 0.05) in the production of IL-1α and IL-8 was detected at the higher concentration of Nutroxsun^®^, lowering the cytokine levels beneath those of the non-irradiated control cells (gray bars). Additionally, Nutroxsun^®^ at the lower concentration (0.01%) completely inhibited (*p* < 0.01) the production of IL-6 in response to UVB radiation, indicating a higher efficacy in reducing IL-6 levels (Figure 5).

### 2.6. Placebo-Controlled Clinical Assessment Results

In the present study, a crossover intervention was conducted in order to decrease the variability between subjects and improve data comparability. Each subject received treatment after an appropriate washout period. A total of 20 subjects were successfully randomized. None of the subjects were lost to follow-up, and all of them were included in the statistical analysis. The population comprised men and women between 18 and 69 years old. Demographic and baseline characteristics are indicated in Table 2. Both the active and the placebo products were well tolerated, and no adverse reactions were reported during the study. The MED measured among subjects ranged from 14.2 to 39.1 mJ/cm^2^ (equivalent to 1.4–3.9 SED, where 1 SED is 100 J/m^2^ erythema weighted UVR).

**Figure 6:**
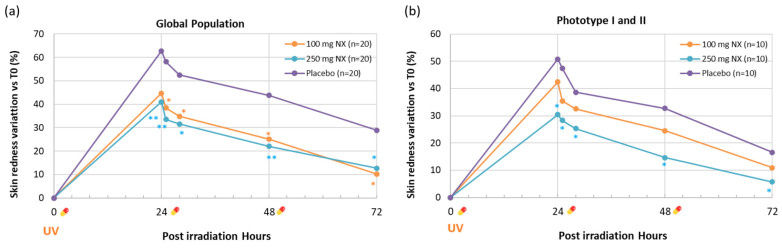
Percentage variation vs. 0 h of skin redness in the placebo (green line), 100 mg (orange line), and 250 mg (blue line) Nutroxsun^®^ (Nx) treatment groups. Results are shown for the global panel (**a**) and the subgroup of subjects with light phototypes (**b**). 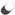 product intake. Statistically significant vs. placebo is reported as * *p* < 0.05 and ** *p* < 0.01.

Erythema is the most noticeable immediate effect of UVR exposure on the skin. It is a sign of the typical inflammatory reaction seen in sunburns [39]. Erythema is observed as a visible redness of the skin due to increased blood volume in the surface and deep dermal vessels. It usually appears 3–5 h after exposure to UVB radiation, peaks at 12–24 h, and gradually fades over 72 h [40]. The effect of Nutroxsun^®^ on UVB-induced skin redness is shown in Table 3 and Figure 6.

Starting conditions were homogenous among the groups (*p* = 0.989). Furthermore, 24 h after UVB exposure to 1 MED, skin redness increased across all treatment groups, indicating that UVB exposure elicited an erythemal response in all groups. However, the severity of this response was mitigated in the group that took Nutroxsun^®^ one hour before UVB exposure. In the placebo group, skin redness increased by 62.7%, while in the Nutroxsun^®^ groups, redness increased by only 44.7% and 41.0% in the 100 mg and 250 mg doses, respectively. After 24 h, the skin redness significantly reduced compared to the placebo group in subjects consuming 250 mg of Nutroxsun^®^ (*p* = 0.006) and after 25 h in subjects taking 100 mg (*p* = 0.02). At 72 h post-UVB exposure, the skin redness had largely resolved in both Nutroxsun^®^ groups. In the 100 mg group, redness was only 10.3% higher than baseline and 12.7% higher in the 250 mg group. No significant differences were detected in skin redness between the start of the study and 72 h (*p* = 0.185 and *p* = 0.07 for the 100 mg and 250 mg groups, respectively). In the placebo-treated group, skin redness remained slightly higher compared to its basal value (*p* < 0.001), indicating delayed recovery compared to the Nutroxsun^®^-treated groups (Table 3 and Figure 6a).

The subgroup analysis of participants with skin phototypes I and II, who are more susceptible to UV-induced erythema, revealed distinct responses to the different doses of Nutroxsun^®^ (Table 3 and Figure 6b). The 250 mg dose was notably more effective than the 100 mg dose, resulting in less skin redness at each time point and a faster recovery compared to the placebo. The placebo group showed the highest increase in redness in this subgroup, with a +50.8% increase at 24 h, which remained elevated throughout the study period (+32.7% at 48 h and +16.6% at 72 h). In participants taking the 100 mg dose, skin redness increased by 42.4% at 24 h (from 8.5 ± 0.4 to 12.0 ± 0.6), which was lower than in the placebo group. Redness progressively decreased over time, with a +24.5% increase at 48 h and +10.9% at 72 h, suggesting a possible protective effect. However, no statistical significances were observed compared to the placebo at any of the time points, probably due to the low number of subjects.

## 3. Discussion

The results from the present study reinforce and expand the photoprotective and anti-photoaging properties previously documented for Nutroxsun^®^, a combination of rosemary and grapefruit extracts rich in polyphenols [25,26]. In the present study, it has been shown that this blend can help mitigate UV-induced oxidative damage, inflammation, and extracellular matrix degradation. These effects are pertinent to both short-term responses, such as erythema, and long-term photoaging, including collagen degradation and skin elastin reduction.

UV exposure accelerates the generation of reactive oxygen species (ROS), which play a critical role in oxidative stress and subsequent skin damage. The current study revealed a significant dose-dependent reduction in intracellular ROS levels in keratinocytes that were pretreated with Nutroxsun^®^. At higher UVB exposure levels (120 mJ/cm^2^), this ingredient was particularly effective, completely inhibiting ROS production at a concentration of 0.02%. These findings are consistent with previous research [25,26], demonstrating that Nutroxsun^®^ can limit oxidative stress induced by UV exposure in both cellular and human models. The observed reduction in ROS is particularly relevant, as ROS are a major factor driving the increase in MMP levels in aged skin [11,12]. Additionally, ROS are considered inflammatory mediators that activate NF-kB signaling, which regulates the expression of pro-inflammatory cytokines and enzymes such as IL-1, IL-6, IL-8, and COX-2. These substances contribute to inflammation and the degradation of the extracellular matrix [11,12].

The degradation of ECM components, particularly collagen and elastin, primarily due to matrix metalloproteinases (MMPs), plays a central role in photoaging [20]. In this study, Nutroxsun^®^ demonstrated a significant protective effect by countering UV-induced upregulation of MMP-1 and MMP-3, which are critical enzymes responsible for the breakdown of collagen and elastin fibers. The ingredient reduced MMP-1 by 57.2% and showed a dose-dependent inhibition of MMP-3, thereby helping to preserve ECM integrity. Additionally, Nutroxsun^®^ treatment led to increased pro-collagen and elastin secretion in fibroblasts exposed to UV, further supporting its role in ECM protection. By significantly mitigating UV-induced reductions in pro-collagen I and elastin, this botanical combination appears to not only preserve ECM components but also enhance the skin’s resilience to photodamage. These findings are consistent with previous studies indicating that the daily oral intake of Nutroxsun^®^ reduces wrinkle depth and improves both net and gross skin elasticity within 15 days [26]. These beneficial effects may be partly due to the enhanced production of elastin and pro-collagen in dermal fibroblasts, along with MMP inhibition, highlighting the ingredient’s potential to prevent photoaging through ECM preservation and repair.

These findings also align with previous studies on both rosemary and grapefruit extracts. Research has shown that rosemary extract and its active component, rosmarinic acid, support the formation of both early microfibrils and mature elastic fibers in normal human dermal fibroblasts (NHDFs) [41]. UVR can reduce procollagen synthesis by downregulating the TGF-β/Smad signaling pathway. Rosmarinic acid has been shown to accelerate elastic fiber formation by upregulating transforming growth factor β-1 (TGF-β1). Additionally, protective effects against UVA-induced damage were reported by preserving collagen type I fiber formation in human skin fibroblasts [42]. Also, both rosmarinic acid and rosemary extracts have been shown to stimulate collagen type I biosynthesis in osteogenesis imperfecta type I skin fibroblasts [43], further highlighting their potential to support dermal structure and resilience. Furthermore, several studies have shown that rosemary extracts downregulate MMPs and inflammatory cytokines, reducing collagen breakdown and skin inflammation [44,45]. Key compounds, such as carnosic acid, inhibit pathways linked to MMP expression, enhancing the skin’s resilience to UV damage [45]. Also, grapefruit flavones such as naringin have been proven to inhibit UVB-stimulated matrix metalloproteinase (MMP-2, MMP-9, and MMP-13) expression in mouse embryonic fibroblast cells (3T3 cells) [46].

On the other hand, the clinical findings highlight the potential of Nutroxsun^®^ in reducing UV-induced erythema and accelerating recovery, with benefits observed as early as 24 h post-UV exposure in subjects taking a 250 mg dose and after 25 h in those taking 100 mg. Seventy-two (72) hours after UVB exposure, skin redness had largely resolved in both groups. This rapid reduction in erythema underscores the blend’s effect as a dietary supplement for daily photoprotection, where reduced redness and faster recovery times may enhance skin tolerance to UV exposure and potentially lower the risk of chronic photodamage.

Susceptibility to sunburn varies significantly among individuals. Phenotypic characteristics that confer high susceptibility to sunburn include fair skin, blue eyes, and red or blond hair. This increased susceptibility is also a marker of an increased risk of melanoma and nonmelanoma skin cancer [47]. A higher dose of Nutroxsun^®^ (250 mg) seemed to be most effective in individuals with UV-sensitive skin types (Fitzpatrick I and II), with a 30.4% increase in redness at 24 h compared to 50.8% in the placebo group. Redness in the 250 mg-treated group also declined more rapidly, reaching baseline by 72 h. However, given the small sample size per phototype group (*n* = 10), these results should be interpreted with caution, as variability within groups could influence the observed trends. Furthermore, the response in phototype III was more variable, leading to differences in the global group’s overall recovery pattern. Although the crossover design used in this study increased statistical power by minimizing inter-individual variability, larger-scale clinical trials are warranted to confirm these findings across broader and more diverse populations. Future studies with larger stratified sample sizes will be needed to confirm whether the observed phototype-dependent trends are consistent across different populations.

This study builds upon previous findings regarding the potential photoprotective benefits of Nutroxsun^®^ and offers enhanced robustness due to a larger sample size and improved methodology [26]. Notably, administering the blend one hour before UV exposure—compared to 30 min in the prior study—may have contributed to stronger clinical outcomes by enhancing the bioavailability of key active compounds present in the rosemary and grapefruit extracts. Although pharmacokinetic data were not collected in the present trial, previous studies have reported the systemic absorption of carnosic acid [48], rosmarinic acid [49,50], and grapefruit flavones [51,52], with optimal plasma levels typically reached approximately one hour after oral intake rather than after just 30 min. Future investigations evaluating different administration intervals or incorporating direct quantification of these compounds in blood or skin tissues could offer further insights into the relationship between systemic exposure and clinical efficacy.

At the tissue level, sunburn results in vasodilation and dermal edema, which manifest clinically as erythema and swelling, respectively. At the cellular and molecular level, sunburn is characterized by an influx of neutrophils and macrophages into the skin, as well as the release of proinflammatory mediators, including TNF-α and different cytokines [6,7]. UVB-induced inflammation is largely driven by IL-8, which recruits neutrophils to the affected area, resulting in tissue damage, keratinocyte proliferation, and the promotion of angiogenesis and tumor growth, including melanoma [53,54,55]. Therefore, suppressing IL-8 may significantly protect against UVB-induced skin inflammation and potentially against its long-term effects [56]. Also, IL-1 α is a key initiator of the UVR inflammatory process and plays a crucial role in mediating the initial neutrophil response at the site of inflammation [57]. In addition, research has shown that a single, total-body UV exposure that caused a significant sunburn reaction led to a notable increase in circulating IL-6, peaking 12 h after exposure. The data suggest that IL-6 is likely released by keratinocytes after UV exposure and enters the bloodstream, where it acts as a key mediator of the systemic sunburn response, triggering fever and acute phase reactions [58].

Notably, in this study, Nutroxsun^®^ significantly lowered IL-8, IL-6, and IL-1α levels down to baseline levels in UV-exposed keratinocytes, correlating with the observed reduction in erythema. In addition to the in vitro and clinical data presented here, a previous in vivo study performed with this botanical combination also showed that oral administration of Nutroxsun^®^ modulates the expression of biomarkers associated with inflammation [59]. By downregulating these proinflammatory cytokines, the botanical blend not only mitigates the immediate inflammatory response triggered by UV exposure but may also help protect against the long-term risks associated with chronic sun exposure.

It is crucial to emphasize that the results of our study are not intended to suggest a replacement for established photoprotective behaviors. The consistent practice of comprehensive photoprotection, including the regular application of sunscreen, remains fundamental in guarding against both the immediate and long-term consequences of UVR exposure, such as sunburn, photocarcinogenesis, and photoaging [60,61]. Nutroxsun^®^ should be regarded as a complementary strategy that can support, but not replace, these essential protective measures.

## 4. Materials and Methods

### 4.1. Test Product

The test product is a commercially available mixture (1:1) of rosemary and citrus extracts (Nutroxsun^®^, supplied by Monteloeder S.L. through Suannutra, Miguel Servet 16, Elche, Alicante, Spain), obtained from dried rosemary (*Rosmarinus officinalis*) leaves and grapefruits (*Citrus paradisi*) extracts, respectively. Nutroxsun total phenolic standard content is higher than 35 gallic acid equivalents (GAE)/100 g dry weight (dw), as determined by Folin assay [62]. The total rosemary phenolic content is higher than 7% dw, and the total grapefruit flavones content is higher than 20% dw, as described previously [25,26].

To perform the Folin–Ciocalteu assay, 50 µL of the test sample or gallic acid standard was mixed with 250 µL of Folin–Ciocalteu reagent, 500 µL of 20% sodium carbonate, and 4.2 mL of distilled water. The mixture was then incubated for 30 min prior to measuring the absorbance at 750 nm. Nutroxsun^®^ samples were previously dissolved in DMSO (1 mg/mL), while gallic acid standards were prepared in ethanol at various concentrations to generate the calibration curve. Total phenolic content was calculated based on the standard curve and expressed as milligrams of gallic acid equivalents per 100 g of dry weight (mg GAE/100 g dw).

### 4.2. MMP Inhibition Study

MMP inhibitor profiling was determined using the Fluorometric MMP Inhibitor Profiling Kit (Enzo Life Sciences Inc., Farmingdale, NY, USA) according to the manufacturer’s instructions. Briefly, the test compound was dissolved in dimethyl sulfoxide (DMSO) and further diluted in assay buffer (50 mM HEPES, 10 mM CaCl2, 0.05% Brij-35, and pH 7.5), maintaining a final DMSO concentration of 1%. Five MMPs (MMP-1, MMP-2, MMP-3, MMP-8, and MMP-12) were individually incubated in a 96-well plate with three different concentrations of Nutroxsun^®^ for 45 min at 37 °C, followed by the addition of a quenched fluorogenic included in the kit. Afterwards, a pre-warmed universal substrate was added to each well, and fluorescence was measured at one-minute intervals using a fluorometer (excitation at 320 nm, emission at 405 nm) for 10 min. MMP activity was calculated by plotting relative fluorescence units (RFU) against time. The slope of the line through its linear portion was then determined, with the slope used as the measurement of enzyme rate (change in RFU/change in time). Percent MMP inhibition was then calculated as ((Uninhibited Rate − Inhibited Rate)/Uninhibited Rate) × 100. Each condition was assayed in duplicate. The half maximal inhibitory concentration (IC50) for each MMP was calculated using the AAT Bioquest IC50 calculator online tool [63].

### 4.3. Cellular Assays

#### 4.3.1. Cell Cultures

To evaluate the antioxidant and anti-inflammatory properties of Nutroxsun^®^, human spontaneously immortalized keratinocytes (HaCaT) from Cell Lines Service GmbH, CLS (Eppelheim, Germany), were used. The HaCaT cells were grown in Dulbecco’s Modified Eagle Medium (DMEM; Gibco/Thermo Fisher Scientific. Waltham, MA, USA) supplemented with 10% fetal bovine serum (FBS; Sigma-Aldrich, St. Louis, MO, USA) and 1% (*v*/*v*) penicillin-streptomycin (Gibco/Thermo Fisher Scientific, Waltham, MA, USA) in a humid atmosphere with CO_2_ (5% *v*/*v*) at 37 °C. The HaCaT cells were trypsinized every third day and seeded in 96-well microplates Falcon^®^, Corning Inc., Corning, NY, USA) to conduct the study.

For pro-collagen type I, elastin, MMP-1 and MMP-3 protein quantification (ELISA), and confluent normal human dermal fibroblasts (NHDFs) (Promocell, Heidelberg, Germany) were maintained in 175 cm^2^ rectangular canted neck cell culture flasks (Corning, Glendale, AZ, USA) in a humidified atmosphere at 37 °C and 5% CO_2_. Cells were detached using TrypLE Express (Gibco, Waltham, MA, USA) for 5 min at 37 °C and inactivated with DMEM supplemented with 4.5 g/L glucose and 10% FBS (Gibco, Waltham, MA, USA) (hereafter D10 medium). The medium was renewed every 2–3 days, and the cells were passaged when fibroblasts reached 70–80% confluency. For the experiments, the cells were then seeded (8 × 10^3^ cells/well) in 96-well clear flat bottom TC-treated culture microplates (Falcon^®^, Corning Inc., Corning, NY, USA) and allowed to attach to the cell culture plastic by incubation overnight at 37 °C, 5% CO_2_. After attachment, the D10 medium was replaced with DMEM + 0.1% FBS (hereafter D0.1 medium) with or without the test samples. FBS reduction was used to eliminate the influence of undefined components on protein secretion. Experimental conditions were adjusted based on the proteins of interest.

#### 4.3.2. Cell Viability

A standard methyl thiazolyl tetrazolium (MTT) assay was used to measure cell viability and cytotoxicity. This colorimetric assay is based on the reduction of yellow tetrazolium salt (3-(4,5-dimethyl thiazol-2-yl)-2,5-diphenyl tetrazolium bromide or MTT) to purple formazan crystals by metabolically active cells. The viable cells contain NAD(P)H-dependent oxidoreductase enzymes that reduce the MTT to formazan. The insoluble formazan crystals are dissolved using a solubilizing solution, and the resulting colored solution is quantified by measuring the absorbance. The MTT assay was performed following the ECVAM Guidelines as established in the ECVAM Database Service on Alternative Methods to Animal Experimentation (MTT assay protocol nr. 17). For the MTT assay, the cells were cultured overnight at a density of 5000 cells/well. The cells were allowed to attach to the cell culture plastic by incubation overnight at 37 °C, 5% CO_2_. In brief, MTT solution 1:11 was added to each well. The plates were incubated in the refrigerated incubator at 37 °C for 3 h. The MTT solution was removed, and DMSO 100% was added to each well to solubilize formazan crystals prior to absorbance measurements at 570 nm and 620 nm as a reference on a scanning multi-well spectrophotometer.

The concentrations of Nutroxsun^®^ used in the cellular assays were selected based on non-cytotoxic levels determined using an MTT assay, ensuring that cell viability remained above 85%.

- For procollagen type I, elastin, MMP-1 and MMP-3 protein quantification (ELISA), and NHDFs were treated with the test product for 48 h.
- For the antioxidant and anti-inflammatory studies, HaCaT were treated with the test product for 2 h.

The MTT assays were set with 8 technical replicates per condition.

#### 4.3.3. UV Radiation

The irradiation protocols were optimized to induce measurable biological effects while minimizing cytotoxicity, as verified through MTT assays.

In the ELISA studies, NHDFs were exposed to UVB or UVA radiation using a portable exposure unit equipped with LZC-UVB and LZC-UVA lamps (Luzchem, Ottawa, ON, Canada). The UVB lamp emitted radiation within the 280–315 nm spectrum, with its maximum peak at 303 nm, while the UVA lamp operated within the 315–400 nm spectrum, with its maximum peak at 351 nm. Both lamps were calibrated using a Luzchem SPR-4001 spectroradiometer (s/n 047) to ensure accurate and reproducible UV dose delivery in physical units.

For MMP-1 and MMP-3 quantification, the cells were exposed to UVB radiation for 10 s, receiving a total dose of 25 mJ/cm^2^. For the pro-collagen I assay, a total dose of 50 mJ/cm^2^ was administered through two separate UVB exposures of 25 mJ/cm^2^ each, delivered 24 h apart. For elastin measurement, the cells were exposed to UVA radiation for 1 h, receiving a total dose of 10,400 mJ/cm^2^.

In the antioxidant and anti-inflammatory assessments, UVB irradiation was delivered using Vilbert Lourmat Bio-Link BLX-E312 (Fisher Bioblock, Illkirch, France). This apparatus is composed of a closed cavity with six 8 W UV lamps at 312 nm, placed at the ceiling of the cavity. The intensity of emitted light was measured using a microprocessor-controlled RMX-3W radiometer (Vilber Lourmat, Collégien, France) to ensure that the accurate UVB dose was delivered in physical units. For antioxidant evaluation, keratinocytes were exposed to two different dosages, 80 or 120 mJ/cm^2^. For the anti-inflammatory assay, a total dose of 60 mJ/cm^2^ was administered to the keratinocytes.

#### 4.3.4. Quantitation of MMP-1 and MMP-3 in Normal Human Dermal Fibroblasts

Fibroblasts were cultured in D0.1 medium and divided into four experimental groups: (1) Control: untreated cells; (2) Cells treated with Nutroxsun^®^ at different concentrations (0.0005%, 0.0025%, and 0.005%); (3) Control + UVB exposure: untreated cells exposed to UVB (25 mJ/cm^2^); and (4) Treatment + UVB: cells exposed to the test samples at the same concentrations and subjected to UVB (25 mJ/cm^2^).

The cells were pre-incubated with Nutroxsun^®^ for 2.5 h, followed by UVB exposure for the relevant groups. After irradiation, the cells were incubated, with or without the test product at different concentrations, for 48 h at 37 °C with 5% CO_2_. Following the incubation period, supernatants were collected and centrifuged, and the resulting cell-depleted supernatants were used to quantify the secreted proteins. MMP-1 and MMP-3 concentrations were measured using enzyme-linked immunosorbent assay (ELISA) kits: Human Collagenase Type 1 ELISA Kit (NBP2-75890, Novus Biologicals, Centennial, CO, USA) and Human Total MMP-3 Quantikine ELISA Kit (DMP300, R&D Systems, Minneapolis, MN, USA), respectively, following the manufacturer’s instructions. The supernatants were diluted 50-fold for MMP-1 and 5-fold for MMP-3 according to the manufacturer’s recommendations to ensure that the optical density at 450 nm was within the linear range of the standard curve. The ELISA allows the calculation of protein concentration through interpolation using a standard curve made by dilutions of known concentrations of the specific protein in the sample diluent, provided by the kit’s manufacturer. Appropriate regression models were applied to calculate the best fit equations for the data obtained and interpolate the values of the test samples.

The raw levels of MMP-1 (ng/mL) and MMP-3 (ng/mL) were corrected to the raw levels of cell viability (determined by MTT as previously described) for each technical replicate. Normalization to cell viability ensured that observed reductions in MMP secretion were due to Nutroxsun^®^’s inhibitory effects rather than variations in cell number.

Afterwards, protein levels/cell viability were normalized versus the untreated control groups (assigned with the 100%) and graphically represented in percentage. The results represent the average of six measurements.

#### 4.3.5. Quantification of Pro-Collagen I

Fibroblasts were cultured in D0.1 medium and divided into three experimental groups: (1) Control: untreated cells; (2) Control + UVB exposure: untreated cells exposed to two doses of UVB (50 mJ/cm^2^); and (3) Treatment + UVB: cells exposed to the test samples at different concentrations (0.0005%, 0.0025%, and 0.005%) and subjected to two doses of UVB (50 mJ/cm^2^).

The cells were pre-incubated with Nutroxsun^®^ for 2.5 h, followed by UVB exposure for the relevant groups. After irradiation, the cells were incubated, with or without the test product at different concentrations, for 24 h at 37 °C with 5% CO_2_. Then, the irradiation step was repeated, keeping the culture medium with the collagen I accumulated up to that point and turning it back to the cells after exposure to UVR. After this second UVB exposure, the cells were kept in the presence of the products until the next day, with a total incubation period of 48 h. Following the incubation period, the supernatants were collected and centrifuged. Pro-collagen I concentrations were measured using a Human Pro-Collagen I alpha 1 ELISA Kit (ab210966, Abcam, Cambridge, UK). The supernatants were diluted 500-fold according to the manufacturer’s recommendations to ensure that the optical density at 450 nm was within the linear range of the standard curve. The results of collagen I (pg/mL) were corrected to the raw levels of cell viability (determined by MTT as previously described) for each technical replicate. Afterwards, protein levels and cell viability were normalized against the untreated control (assigned with the 100%) and graphically represented in percentage. The results represent the average of six measurements.

#### 4.3.6. Quantification of Elastin

Fibroblasts were cultured in D0.1 medium and divided into three experimental groups: (1) Control: untreated cells; (2) Control + UVA exposure: untreated cells exposed to UVA (10,400 mJ/cm^2^, 1 h); and (3) Treatment + UVA: cells exposed to the test samples at different concentrations (0.0005%, 0.0025%, and 0.005%) and subjected to UVA (10,400 mJ/cm^2^). The cells were pre-incubated with Nutroxsun^®^ for 2.5 h, followed by UVA exposure for the relevant groups. After irradiation, the cells were incubated, with or without the test product at different concentrations, for 24 h at 37 °C with 5% CO_2_ to allow for the accumulation of elastin. After the incubation period, the cell culture supernatants were collected and centrifuged. Elastin concentrations were quantified using the Human Elastin ELISA Kit (ab239433, Abcam, Cambridge, UK) according to the manufacturer’s guidelines, without dilution of the supernatants. The raw levels of elastin (ng/mL) were corrected to the raw levels of cell viability (determined using MTT as previously described) for each technical replicate. Afterwards, elastin levels and cell viability were normalized against untreated control UV-irradiated groups and graphically represented in percentage. The results represent the average of six measurements.

#### 4.3.7. Antioxidant Assessment by UVB-Induced Oxidative Stress

Here, 2′,7′-dichlorofluorescein diacetate (H2DCF-DA, Molecular Probes™, Invitrogen™/Thermo Fisher Scientific, Waltham, MA, USA) was used as a probe to monitor the intracellular ROS generation induced by UVB radiation. Briefly, HaCaT cells were cultured in a 96-well black plate and maintained in medium for 24 h until 90–100% confluence was reached. Then, the cells were treated with Nutroxsun^®^ (0.01% and 0.02%) for 2 h, followed by exposure to UVB light (80 or 120 mJ/m^2^) with Bio-Link Cross-linker BLX-E312 (Vilber Lourmat, France). Afterwards, PBS was replaced with fresh medium, and the cells were incubated with H2DCF-DA (10 µg/mL) for 1 h at 37 °C and 5% CO_2_. Fluorescence was measured using a Cytation 3 Cell Imaging Multimode reader (BioTek Instruments, Winooski, VT, USA) with 485 nm excitation and 535 nm emission filters. Basal antioxidant activity was determined by comparing treated and untreated cells in the absence of UVB radiation and graphically represented in percentage. The results represent the average of six measurements.

#### 4.3.8. Anti-Inflammatory Assessment by UVB-Induced Cytokines

HaCaT cells were cultured in 96-well dishes and maintained in the medium for 24 h. When 70% confluence was reached, the cells were washed with phosphate-buffered saline (PBS) and treated with a thin layer of PBS containing Nutroxsun^®^ at 0.01% or 0.02% 2 h, followed by UVB light irradiation (60 mJ/m^2^) with Bio-Link Cross-linker BLX-E312 (Vilber Lourmat, France). Then, PBS was replaced with fresh medium for a period of 24 h. Finally, cell culture supernatants were collected, and cytokines (IL-1α, IL-8 and IL-6) were measured using a multiplexed assay of fluorescent microspheres (Luminex MAGPIX^®^ Toronto, ON, Canada) following the manufacturer’s instructions. Each condition was assayed in triplicate. The content of different interleukins (pg/mL) were corrected to the raw levels of cell viability (determined using MTT as previously described) for each technical replicate. The results represent the average of six measurements.

#### 4.3.9. Data Processing and Statistical Analysis

Data outliers were identified (IQR method) and excluded from the analysis. Statistically significant differences between the groups were determined using one-way analysis of variance (ANOVA). Multiple comparisons were performed using Tukey’s multiple-comparisons test, and *p*-values < 0.05 were considered statistically significant.

### 4.4. Placebo-Controlled Clinical–Instrumental Assessment

The objective of this study was to assess the efficacy of Nutroxsun^®^ in increasing the response of the skin to UVB exposure. In particular, the efficacy of the product in decreasing UV-induced skin redness was assessed.

#### 4.4.1. Study Design

A monocentric, placebo-controlled, crossover, inter- and intra-group comparison study was performed. The study was conducted in accordance with the World Medical Association’s (WMA) Helsinki Declaration and its amendments at Complife Italia Srl (27028 San Martino Siccomario, PV, Italy) dermatological facilities. Both the study protocol and the informed consent form were approved by the “Comitato Etico Indipendente per le Indagini Cliniche Non Farmacologiche”. A signed informed consent form was obtained from all the subjects participating in the study before the study took place.

#### 4.4.2. Subjects

In total, 20 male and female adult subjects with skin phototypes from I to III according to Fitzpatrick classification [64] were enrolled. The subjects were in general good health, with no eating disorders or a history of metabolic syndrome. Key inclusion criteria included having a test area on their backs, uniform in color, free from nevi, blemishes, solar lentigo, and hair, and no sun exposure to the back area for at least two months prior to the study. Additionally, the subjects were required to avoid any UV exposure (artificial UV light or sunlight) throughout the study period. Exclusion criteria included pregnancy or intention to become pregnant, lactation, food intolerances/allergies, pharmacological treatments known to interfere with the test product or affect metabolism, participation in another similar study, and unwillingness or inability to comply with the study protocol requirements. Subjects using food supplements with active ingredients that might influence skin response to UV rays or skin aging were also excluded. All inclusion and exclusion criteria were verified through a questionnaire during the screening visit. During the entire study period, the subjects were also asked to maintain their usual dietary habits. Withdrawn, lost to follow-up, or drop-out subjects were not replaced.

#### 4.4.3. Intervention

The subjects were randomly assigned to receive 100 mg Nutroxsun^®^, 250 mg Nutroxsun^®^—which aligns with its intended use as a dietary supplement—or a placebo product.

In the low-dose group, each jelly capsule contained 100 mg of Nutroxsun, 250 mg of maltodextrin, 2 mg of yellow iron oxide, and 98 mg of gelatin. In the high-dose group, each capsule contained 250 mg of Nutroxsun, 100 mg of maltodextrin, 2 mg of yellow iron oxide, and 98 mg of gelatin. The placebo product consisted of a jelly capsule containing 350 mg of maltodextrin, 2 mg of yellow iron oxide, and 98 mg of gelatin.

**Figure 7:**
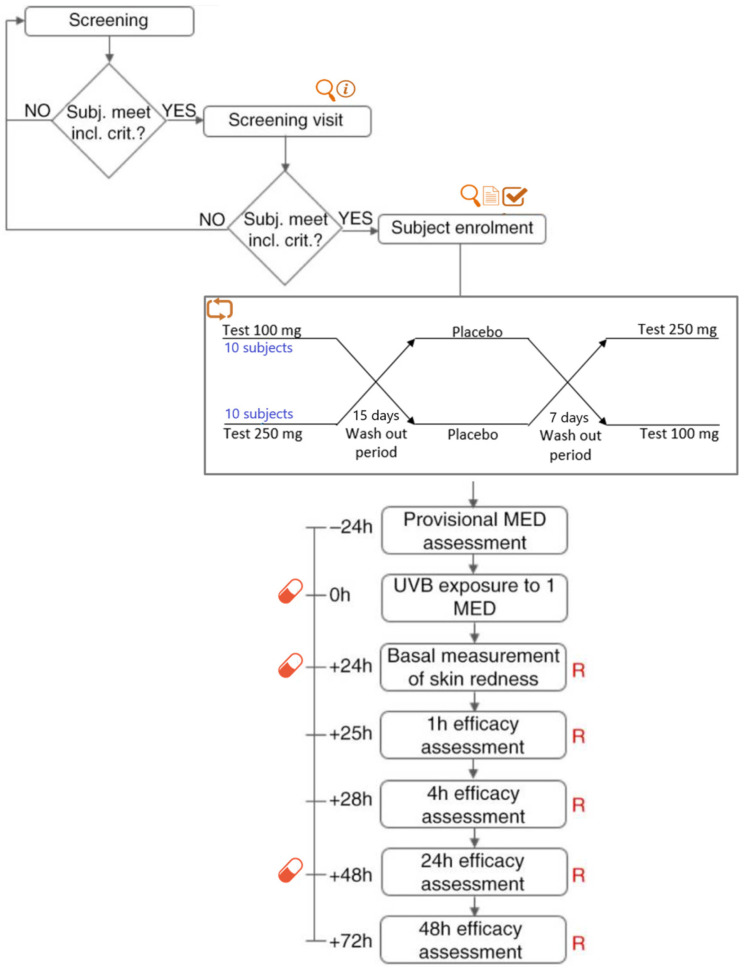
Study flow and design. Subjects who met the inclusion criteria were then screened by a dermatologist. During the screening visit, a physical examination was carried out to assess the uniformity of the test area (back). Subjects meeting the inclusion criteria were then enrolled and randomized to participate in the study. Legends: 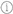 information, 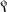 physical examination, 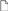 informed consent signature, 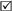 eligibility check, 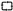 randomization, R skin redness, 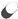 product intake.

The study was designed as a crossover trial in which each subject received each treatment after a washout period to prevent carryover effects between treatments. The subjects received the first dose (either 100 mg or 250 mg) of the test product or the placebo 1 h before UVB exposure to 1 MED. Supplemental doses were given 24 and 48 h after UV exposure to mimic typical daily supplementation (Figure 7).

#### 4.4.4. Sample Size

The sample size of 20 participants was determined based on data from a preliminary crossover study with 5 individuals, which demonstrated a 15% reduction in skin redness (a* value) following Nutroxsun^®^ treatment compared to placebo. Using an estimated effect size, 80% power, and a 5% significance level, the sample size was calculated to detect significant differences between the treatments. The crossover design further enhanced statistical power by minimizing inter-individual variability, making 20 participants sufficient for the study’s objectives.

#### 4.4.5. Randomization and Blinding

Eligible subjects were assigned to the different groups using PASS 11 statistical software (version 11.0.8; PASS, LLC. Kaysville, UT, USA), with randomization restricted (Random Sorting, maximum allowable % deviation = 10%). The software was run on Windows Server 2008 R2 Standard SP1. The subjects, investigators, and collaborators were blinded to product assignment. The dietary supplement and placebo products were in opaque, colored capsules with identical appearances. Sequentially numbered, opaque, and sealed envelopes reporting the unblinded treatment allocation were prepared for each subject.

#### 4.4.6. Efficacy Evaluation. Skin Redness Recovery

One day before the study started, a provisional Minimal Erythema Dose (MED) was determined for each participant to center the UV dose ranges for the skin redness recovery assessment. A series of UV doses (geometric progression of 1.12×) were applied on 6 small subsites of the skin of the back region between the scapula line and the waist. The total area of the individual test sites was 40 cm^2^ and there was a minimum distance of 1 cm between the borders of adjacent test sites to ensure no overlap. These subsites were distinct from the main test area used later. The source of UV radiation was a Multiport 300–601 W Solar simulator (Solar^®^ Light Co. Inc., Philadelphia, PA, USA) compliant with ISO 24444:2019 standard requirements [65]. The UV dose was adjusted with a model PMA 2100 radiometer, which measures erythema-effective radiation, equipped with a PMA 2103 LLG SUV detector (Solar^®^ Light Co. Inc., Philadelphia, PA, USA). The solar simulator is designed to emit an appropriate radiometric proportion of UVA and UVB within the spectrum (290–400 nm). Specifically, it ensures that the UVA II irradiance (320–340 nm) accounts for at least 20% of the total UV irradiance, while the UVA I irradiance (340–400 nm) exceeds 60%.

Then, 20 ± 4 h after UV exposure, a minimal erythema dose (MED) was assessed, under blind conditions, by an experienced technician in a room with matt neutral wall color and sufficient illumination conditions (at least 450 lux). MED assessment was considered invalid if (i) the series of UVB exposures on a subject failed to elicit an erythemal response on any sub-site, (ii) all sub-sites in the exposure series showed an erythemal response, and (iii) erythemal responses within an exposure series were randomly absent. The final MED determined for each participant was subsequently used to individualize the UV dose applied during the main intervention.

Skin redness in the UV-exposed site (1 MED) was measured using a colorimeter/spectrophotometer CM-700D (Konica Minolta, Milan, Italy). The parameter measured was the a* value in the CIELAB chromatic space [66], which correlates with skin redness. Measurements were taken excluding specular reflection at baseline (before UV exposure) and 24, 25, 48, and 72 h post-irradiation. Each subject’s measurements were taken by the same trained researcher to minimize variability.

#### 4.4.7. Safety

The occurrence of adverse events (AEs) was monitored throughout the study by the investigators and based on the subjects’ diary entries. The investigators rated the observed and reported AEs as being either severe or non-severe based on their potential relationship to study treatment.

#### 4.4.8. Statistical Analysis and Analytical Plan

Efficacy and safety analysis was based on the per protocol population and safety population, respectively. Intragroup (vs. baseline) statistical analysis was carried out using repeated measures analysis of variance (RM-ANOVA), followed by the Tukey–Kramer post-test. Intergroup (between treatments) statistical analysis was carried out using a multivariate analysis of variance (M-ANOVA). A *p*-value < 0.05 was considered statistically significant. Statistical analysis output was reported as follows: * *p* < 0.05, ** *p* < 0.01, and *** *p* < 0.001.

## 5. Conclusions

This study supports Nutroxsun^®^, a formulation combining grapefruit and rosemary extracts, as a promising agent for oral photoprotection. It was evaluated for its ability to inhibit ROS production, downregulate inflammatory cytokines, and preserve essential ECM components such as collagen and elastin in vitro, thereby providing a mechanistic basis for its photoprotective effects. The clinical results further indicated that the ingredient effectively reduces UV-induced skin redness after UV exposure. Overall, these findings highlight the potential of Nutroxsun^®^ as a comprehensive strategy to mitigate UV-induced skin damage and photoaging.

## Figures and Tables

**Table 1:** MMPs evaluated and their substrates [16,20].

Metalloproteinases		Substrates
MMP-1	Interstitial collagenase	Collagen I, II, III, VII, VIII, IX, gelatin, etaktyn, aggrecan
MMP-2	Gelatinase A	Collagen I, IV, V, VII, X, XI, XIV, gelatin, fibronectin, laminin, agrecan, elastin
MMP-3	Stromelysin 1	Collagen III, IV, V, IX, X, XI, gelatin, laminin, fibronectin, elastin, agrecan, cazein, tenascin
MMP-8	Neutrophil collagenase	Collagen types I, III, gelatin, fibronectin
MMP-12	Macrophage elastase	Elastin, fibronectin, laminin, collagen types IV, V, gelatin, casein

**Table 2:** Demographic characteristics.

	Panel	Units
**Sex**		
Male	5	No.
Female	15	No.
**Skin phototypes**		
I	10%	%
II	40%	%
III	50%	%
**Age**	45.1 ± 3.1	years
**Skin erythema (basal)**	8.1 ± 0.3	a.u.

**Table 3:** Skin redness time course after 1 MED UVB exposure in the global panel.

Global Panel	0 h	24 h	25 h	28 h	48 h	72 h
100 mg NX (*n* = 20)	8.1 ± 0.3	11.7 ± 0.5	11.2 ± 0.4	11.0 ± 0.5	10.2 ± 0.5	9.0 ± 0.5
		(+44.7%)	(+38.4% *)	(+34.8% *)	(+25.1% *)	(+10.3% *)
250 mg NX (*n* = 20)	8.3 ± 0.3	11.5 ± 0.4	11.0 ± 0.4	10.8 ± 0.5	10.1 ± 0.4	9.3 ± 0.5
		(+41.0% **)	(+33.5% **)	(+31.6% *)	(+22.0% **)	(+12.7% *)
Placebo (*n* = 20)	8.0 ± 0.4	12.4 ± 0.2	12.1 ± 0.2	11.7 ± 0.2	11.0 ± 0.2	9.9 ± 0.2
		(+62.7%)	(+58.1%)	(+52.5%)	(+43.8%)	(+28.9%)
**Phototype I and II subgroup**						
100 mg NX (*n* = 10)	8.5 ± 0.4	12.0 ± 0.6	11.5 ± 0.5	11.2 ± 0.6	10.6 ± 0.6	9.5 ± 0.6
		(+42.4%)	(+35.4%)	(+32.5%)	(+24.5%)	(+10.9%)
250 mg NX (*n* =10)	8.8 ± 0.4	11.3 ± 0.5	11.1 ± 0.4	10.8 ± 0.5	9.9 ± 0.4	9.2 ± 0.5
		(+30.4% *)	(+28.3% *)	(+25.3% *)	(+14.6% *)	(+5.7% *)
Placebo (*n* = 10)	8.5 ± 0.6	12.4 ± 0.4	12.1 ± 0.4	11.4 ± 0.3	11.0 ± 0.3	9.7 ± 0.3
		(+50.8%)	(+47.4%)	(+38.6%)	(+32.7%)	(+16.6%)

Data are reported as mean value ± SEM in arbitrary units (a.u.). In parentheses, the percentage variation vs. 0 h is reported. Statistically significant vs. placebo is reported as * *p* < 0.05 and ** *p* < 0.01.

## Data Availability

Data may be available upon request to the corresponding author.
